# Combining Asset Accumulation and Multifamily Group Intervention to Improve Mental Health for Adolescent Girls: A Cluster-Randomized Trial in Uganda

**DOI:** 10.1016/j.jadohealth.2023.08.012

**Published:** 2023-09-16

**Authors:** Leyla Karimli, Proscovia Nabunya, Fred M. Ssewamala, Darejan Dvalishvili

**Affiliations:** a Social Welfare Department, Luskin School of Public Affairs, University of California, Los Angeles (UCLA), Los Angeles, California; b International Center for Child Health and Development, Brown School of Social Work, Washington University in St. Louis, St. Louis, Missouri

**Keywords:** Family functioning and adolescent mental health, Moderator analyses, Family functioning as a moderator of the intervention effect on adolescent mental health, Sub-Saharan Africa, Cluster-randomized controlled trial, Mental health of adolescent girls, Family cohesion, Quality of child-parent relationship, Family communication, Beck Hopelessness Scale, Tennessee Self-Concept Scale, Rosenberg Self-Esteem Scale, Depression

## Abstract

**Purpose:**

The aim of this study is to expand the current knowledge on the relationship between poverty, family functioning, and the mental health of adolescent girls in families affected by poverty and HIV/AIDS in southern Uganda. The study investigates the association between family functioning and mental health and examines whether family functioning moderates the intervention effect on adolescent mental health.

**Methods:**

Longitudinal data were collected over the course of 24 months in a cluster randomized controlled trial conducted among N=1,260 girls aged 14–17 years in Uganda. Participants were randomized into control group (n=408 girls from n=16 schools), matched youth development accounts treatment, YDA (n=471 girls from n=16 schools), and integrated intervention combining YDA with multiple family group component (n=381 girls from n=15 schools).

**Results:**

We found a significant positive association between family functioning and mental health of adolescent girls in our sample. Moderator analyses suggests that effect of the intervention on Beck Hopelessness Scale was significantly moderated by family cohesion $\left(\chi^{2}\left(4\right)=21.43;p=\;.000\right)$, frequency of family communication $\left(\chi^{2}\left(4\right)=9.65;p=\;.047\right)$, and quality of child-caregiver relationship $\left(\chi^{2}\left(4\right)=11.12;p=\;.025\right)$. Additionally, the intervention effect on depression was moderated by the comfort of family communication $\left(\chi^{2}(4)=10.2;\;p=\;.037\right)$.

**Discussion:**

The study findings highlight the importance of family functioning when examining the link from poverty to adolescent mental health. The study contributes to the scarce evidence suggesting that asset-accumulation opportunities combined with a family strengthening component may improve parenting practices and adolescent mental health in poor households.

In sub-Saharan Africa, adolescents (aged 10–19 years) constituting 23% of the total population face an elevated risk of mental health disorders, along with a heightened risk of exposure to poverty and associated strains to family functioning [1]. According to the most recent systematic review of adolescent mental health in SSA [2], more than one in four adolescents in the region experience depression, almost one in three adolescents has anxiety disorders, 41% exhibit emotional and behavioral problems, and more than one in five adolescents struggle with post-traumatic stress disorder. In Uganda, depression is prevalent in 23.6% of cases [3].

The high prevalence of mental health disorders among adolescents in sub-Saharan Africa is attributed to a range of often interconnected stressors, including poverty, exposure to violence, abuse and neglect, the detrimental effect of HIV, and inadequate health systems [2]. Poverty—one of the main risk factors for suboptimal mental health in adolescents—diminishes families’ ability to care for children, negatively affects parent-child communication [4], and impairs family functioning referring to the family’s capacity to meet the physical, emotional, and social needs of its members and achieve their overall well-being [5]. This complexity is further compounded by gender dynamics as, due to gender disparities in exposure to the above-mentioned stressors, adolescent girls are at higher risk of experiencing adverse mental health outcomes than their male counterparts [6].

The link between poverty, family functioning, and adolescent mental health has been examined within a theoretical framework that draws upon the social causation argument [7], the Family Stress Model [8], and Asset Theory [9]. The social causation argument [7] suggests that poverty and economic deprivation lead to demographic disadvantages, such as discrimination, environmental adversity, and stressful life events that are accountable for psychological distress and mental health disorders at both individual and family levels. The Family Stress Model [8,10] focuses on the impact of stressors and adversities, including those related to financial strains, on family functioning and the well-being of individual family members. It posits that material deprivation and hardships can create economic pressure on parents, resulting in emotional distress and disruptive parenting. This, in turn, increases family conflict and impairs family functioning, thus exacerbating adolescent’s internalizing and externalizing symptoms and disruptive behavior disorders. Asset Theory [9] highlights the importance of asset accumulation and wealth creation in low-income settings. It posits that asset ownership can have significant positive effect on individuals’ well-being, including enhanced financial security, improved educational attainment and health outcomes, and increased social participation. The theory proposes that by accumulating assets, individuals and families in low-income settings develop a greater sense of agency. It informs programs and policies aimed at mitigating the adverse effect of economic hardships by suggesting that accumulation of economic assets, such as savings and income generating activities, can reduce exposure to socioeconomic stressors, thus leading to improved family functioning and psychosocial well-being.

Empirical evidence suggests that a strong family functioning is among the most crucial relational factors that can effectively mitigate the impact of poverty on the mental health of adolescents [11]. Specifically, family cohesion, family support, and the quality of family communication plays a significant role in determining the parent–child relationship, which, in turn, affects adolescents’ psychosocial well-being. Research has shown that open communication between parents and children can act as a protective factor against psychological and behavioral problems in young people [12]. Family cohesion and daily parent-child interactions, involvement, and discussions around role expectations are also essential for adolescent development [13]. Conversely, families that spend disproportionate amounts of time trying to acquire material and financial resources for their households may have less frequent parent-child communication and interaction—leading to suboptimal psychological adjustment and poor mental health outcomes among adolescents [14].

Numerous studies on adolescent mental health highlight the importance of enhancing family functioning and improving parenting skills through interventions that include parent/caregiver-training, psychoeducation, behavioral theory, and quality of life therapy [15]. Some of these studies have indicated that multifamily group (MFG) therapy—a combination of family and group therapy, where families with similar experiences are provided an opportunity to communicate in a safe setting—can be a valuable approach to addressing adolescent mental health [16]. MFG therapy is a form of a group therapy that focuses on addressing the needs of individual families within the context of a larger group setting. This larger group functions as a system where each member and family unit serve specific roles. The clinical process consists of distinct phases, i.e., beginning, middle, and ending. The focus is on therapeutic processes, including child management practices, emotional regulation, and development of new behaviors tailored to the needs of families within the group setting [17]. In the most recent systematic review of preventive psychological interventions focused on children of parents with mental illness, MFG therapy—incorporating psychoeducation, emotional regulation, parenting skills, and peer support—was found to be the most efficient in terms of improving family dynamics and parental skills [18]. Therapy models reviewed here employ the MFG approach and therapeutic processes that target family dynamics to tackle issues such as poor parent-child interactions, family stress, severe or permissive parenting styles, and stigmatization or isolation. In another study, a systematic review and meta-analyses of MFGT for families affected by eating disorders found mixed evidence regarding its effect on strengthening family functioning or improved caregiving skills [19].The multifamily group therapy examined in this review includes interventions designed to reduce perceived isolation and stigma, improve family relationships, and facilitate family skill building. This therapeutic approach offers comprehensive support that targets both patient-specific and family-related factors.

A separate body of research focusing on social determinants of adolescent mental health emphasizes the significance of addressing poverty reduction and implementing asset accumulation interventions to improve adolescent mental health [20–22]. Asset accumulation interventions where adolescents are direct and explicit recipients of the treatment were shown to have significant positive effect on adolescent mental health [20,21]. These interventions, known as Youth Development Accounts (YDAs) or Child Savings Accounts, have also been linked to reduced parental stress and improved family communication [23–26]. Thus, by addressing poverty, which is a common stressor for both adolescent mental health and family functioning, these interventions improve adolescent well-being as well as the overall family dynamics. On the other hand, limited studies examining integrated interventions that combine poverty reduction activities with a separate family strengthening component suggest that significant improvements in parenting practices are observed only among participants who receive combined (poverty reduction and family strengthening) treatment, as opposed to receiving poverty reduction intervention alone [27]. Moreover, systematic reviews and meta-analyses synthesizing effect of poverty reduction interventions on adolescent mental health [28,29] report inconclusive results, contingent upon underlying factors, including the family environment. This evidence, albeit limited, suggests that integrating a family strengthening component into poverty reduction interventions may be more effective in achieving lasting and significant impact on adolescent mental health.

To enhance the existing body of evidence and foster a deeper understanding of the intersection between poverty, family functioning, and mental health of adolescent girls in families impacted by poverty and HIV/AIDS in southern Uganda we examine the following three questions informed by the theoretical framework outlined above:

1. Does asset accumulation intervention, both in isolation and when combined with MFG component, improve family functioning as reported by adolescent girls?
2. Does enhanced family functioning correlate with improved mental health of adolescent girls over the 24-month study period? and
3. Does family functioning moderate (i.e., affect the direction and/or strength of) the intervention effect on mental health of adolescent girls? Direct effects of the intervention on adolescent mental health were reported elsewhere [21,30].

## Methods

### Study design and sampling

The study uses data collected from 1,260 adolescent girls in a three-arm randomized controlled trial funded by the National Institute of Mental Health. To be included in the study, participants had to be: 1) female; 2) age 14–17 years; 3) enrolled in the first year of secondary school in one of the five districts in Southern Uganda (i.e., Rakai, Kyotera, Masaka, Lwengo, Kalungu); and 4) living in a family (not in institutions, orphanages, or streets). Participants were identified using recruitment procedures validated in prior studies conducted in the area [31]. In coordination with the school administration, school enrollment procedures were used to identify and contact eligible caregivers. Flyers were distributed to caregivers, notifying them about the study and inviting them to meet the in-country project coordinator for individual informational sessions. These meetings served to inform caregivers and adolescents about the study’s objectives, voluntary participation, extent of involvement, associated risks and benefits, as well as protection and confidentiality measures.

Participants randomized to the usual care condition received standard adolescent sexual and reproductive health sexual education provided to all secondary school students in Uganda. The curriculum covers topics related to risk taking behaviors, such as substance use, sexual risk behaviors and delaying sex, safer sex practices and contraception, sexual risk possibility situations and ways of avoiding them, gender equality and delaying marriage.

Participants in treatment arm 1 (hereafter YDA) were enrolled in a savings program at formal financial institution (bank) with savings being matched on a 1:1 rate. The bank account was opened in the name of adolescent, and, in compliance with Uganda law, adolescent’s primary caregiver had to be a co-signed until adolescent reaches 18 years of age. The matching funds were intended to cover costs of adolescent’s education and skill training. Participant’s access to the matching funds were conditional upon completion of 12 financial management workshops over 12 months offered by the community agency (Reach the Youth-Uganda) in collaboration with banks holding the YDAs and covering basic principles of financial management.

Participants in treatment arm 2 (hereafter YDA + MFG) received an MFG family-strengthening intervention in addition to being enrolled in YDAs described above. The MFG intervention was offered as a 16-week (one session per week) family dialogue and family strengthening curriculum delivered by a trained community health worker and a trained peer parent under the supervision of a project staff. Each session lasted for 45–60 minutes and involved a group of 12–20 families. The intervention targets family processes referred to as the 4 Rs (Rules, Responsibility, Relationships, and Respectful Communication) and 2Ss (Stress and Social Support). Sessions covered topics related to behavioral health knowledge, identifying and building family support system, strengthening family communication, and mobilizing family resources in response to environmental stressors. The intervention was adapted to the context of Uganda [30].

Randomization was conducted to school level (n = 42 schools) to avoid cross-contamination. First, stratified random sampling was used to assign schools to four strata based on student population size (medium vs. large) and geographical location (urban vs. rural). Second, the restricted randomization technique was used within the four strata to randomly assign each of the 42 schools to one of the three study arms. The attrition rate at 12 months was 3.3%, with 41 participants dropping out of the study. Additionally, at 24 months, 54 participants dropped out of the study, resulting in a total attrition rate of 7.5% (see CONSORT chart attached). No discernible patterns or systematic factors appear to be associated with the attrition. Consequently, we consider this as missing completely at random and employ listwise complete case analysis to handle the missing data.

The interventions were administered over a 24-month period, with data collected at baseline (preintervention), during intervention (12-months) and 24-months follow-up (after the intervention). Given the number of schools receiving the treatment (n = 31), the process of intervention delivery was staggered. Specifically, the intervention was delivered in a set of schools at a time. Once this was complete, the team moved on to the next set of schools. For schools randomized to treatment arm 2, the YDA intervention component was administered continuously for 24 months. As such, follow-up data were collected at 12 and 24-months postintervention initiation to allow schools equal amount of time for treatment and follow-up. A 90-minute structured survey was administered by Ugandan interviewers who were trained on Good Clinical Practice and obtained the Collaborative Institutional Training Initiative Certificate before interacting with study participants. Voluntary written informed assent and consent were obtained from adolescent participants as well as their caregivers, respectively. The study protocol was approved by the Washington University in St. Louis Review Board (#201703102) and by in-country local IRBs in Uganda: Uganda Virus Research Institute (UVRI–GC/127/17/07/619), and Uganda National Council of Science and Technology (UNCST–SS4406). The study protocol is registered at Clinicaltrial.gov (ID# NCT03307226).

### Measures

The main focus of the study is to investigate adolescent mental health as the primary outcome. As secondary outcomes, the study also examines family functioning. All the measures employed in this study have been rigorously tested and validated with adolescents in Uganda [20,32,33].

#### Adolescent mental health.

To assess mental health of study participants, we use four measures described below:

1. Beck Depression Inventory [34] is a 21-item scale measuring adolescents’ depressive symptoms. Each item has four response options that correspond to different levels of symptomology for clinical depression. The scale ranges from 0 to 63, with higher scores indicating higher levels of depression. The scale has high internal reliability (Cronbach’s alpha ranging from 0.83 at baseline to 0.81 at 24-month follow-up).
2. Tennessee Self-Concept Scale [35] measures adolescents’ sense of identity and self-satisfaction using 20 items. The scale ranges from 0 to 80, with higher scores indicting higher levels of adolescents’ self-concept. The scale has high internal reliability (Cronbach’s alpha ranges from is 0.83 at baseline to 0.86 at 24-month follow-up)
3. Rosenberg Self-Esteem [36] is a 10-item scale that measures adolescents’ self-image. Scale, ranging from 0 to 30, has high internal reliability (Cronbach’s alpha ranges from is 0.77 at baseline to 0.71 at 24-month follow-up)
4. Beck Hopelessness Scale [37] is a 20-item scale that captures adolescent’s hopelessness and pessimistic attitudes toward the future. The scale, consisting of binary items, ranges from 0 to 20, with higher scores corresponding to higher levels of hopelessness. The scale has high internal reliability (Cronbach’s alpha ranges from is 0.71 at baseline to 0.74 at 24-month follow-up).

#### Family functioning.

Consistent with other studies [7], we use four measures adapted from the Family Environment Scale [38], the Family Assessment Measure [39], and the Social Support Behavior Scale (SS-B) [40], to assess family functioning, as described below:

1. *Family cohesion* is a composite score of seven items that captures adolescent’s report on whether family members ask each other for help, spend time with each other and do things together, feel close to each other, and are available for each other to talk and to listen. Each of the seven items was assessed using a five-point Likert scale ranging from 0 (never) to 4 (always). The composite score ranges from 0 to 28, with higher scores reflecting higher family cohesion. The scale has high internal reliability (Cronbach’s alpha ranges from 0.72 at baseline to 0.75 at 24-month follow-up).
2. *Frequency of family communication* is a composite score of 10 items reflecting how frequently adolescents communicate with their caregivers discussing specific topics, such as, risk-taking behaviors, HIV/AIDS, education, and future planning. A five-point Liker scale, ranging from 0 (never) to 4 (always), was used to assess each of the 10 items. The composite score ranges from 0 to 40. Higher scores reflect higher frequency of communication. The scale has high internal reliability (Cronbach’s alpha ranges from 0.81 at baseline to 0.83 at 24-month follow-up).
3. *Comfort of family communication* is a composite score of 10 items reflecting how comfortable adolescents feel communicating with their caregivers around specific topics, including risk-taking behaviors, HIV/AIDS, education, and future planning. 10 items were assessed using a five-point Likert scale, with scores ranging from 0 (very uncomfortable) to 4 (very comfortable). The composite score ranges from 0 to 30, with higher scores reflecting greater level of comfort in communication. The scale has high internal reliability (Cronbach’s alpha ranges from 0.87 at baseline to 0.85 at 24-month follow-up).
4. *Child-caregiver relationship* is a composite score that consists of 14 items measuring the extent to which the adolescent perceives the caregiver as involved in their life (acceptance and warmth); and the extent to which the caregiver employs a noncoercive, democratic discipline and encourages the adolescent to express individuality within the family (psychological autonomy). Each of the 14 items was measured on a five-point Likert scale ranging from 0 (never) to 4 (always). The score ranges from 0 to 56 with higher scores reflecting higher quality of child-caregiver relationship. The scale has high internal reliability (Cronbach’s alpha ranges from 0.83 at baseline to 0.83 at 24-month follow-up).

### Statistical analyses procedures

To report baseline characteristics of our sample in accordance with the CONSORT guidelines, we examine baseline differences across the three study arms. Due to the nested nature of our data (i.e., individuals nested in schools), we report adjusted Wald statistics that shows individual-level variations and accounts for potential school-level correlations.

To examine the intervention effect on family functioning, we ran separate multilevel mixed-effects models for each of the four measures of the family functioning. All models allow for between-school variability and within-individual variability as a random effect (i.e., random intercepts at individual and school levels, using the <mixed> command in Stata 17). Models estimate subject-specific effects, taking into account the school-level clustering and potential within-individual correlations [41]. This addresses the risk of potential correlation for within-school observations, which is due to randomization at the school level. We decompose effects to obtain marginal treatment effect for each treatment group at each time point, as well as compare treatment groups to each other at each time point. Our models were run according to the specification below:

(1) $$Y_{it}=\alpha_{0}+\beta_{1}I_{i}+\beta_{2}T_{it}+\beta_{3}\left(I_{i}*T_{it}\right)+u_{s}+e_{t}+z_{i}$$

where $Y_{it}$ is the continuous outcome (i.e., family functioning measure) for the i-th observations (i=1,2,….1260) at time t(t=1,2,3); I is treatment (I=0 for control group; I=1 for treatment arm YDA; I=2 for treatment arm YDA + MFG); T is time (T=1 at baseline; T=2 at 12 months; and T=3 at 24 months); $u_{s}$ is the level 1 error (i.e. differences between the expected and observed values of outcome at school level); $e_{t}$ is the level 2 error (i.e. difference between the expected and observed values of outcome at time level); and $z_{i}$ is the level 3 error (i.e. difference between the expected and observed values of outcome at individual level). To account for school-level clustering in estimation of subject-specific effects [42], the model adjusts robust standard errors for clustering within villages. To report the treatment effect, we report time-within-group simple effect comparisons obtained through multiple pairwise comparisons. As a precaution for false discovery, we use Sidak’s adjustment method, which is a conservative method designed to provide p value corrections due to multiple comparisons [43]. It protects from potential inflation of alpha level, which could lead to “Type I” error (i.e., rejecting the null hypothesis when it is true), thus minimizing the risk of false discovery.

To examine how family functioning relate to adolescent mental health over the course of the study, we use the hierarchical variable entry (or hierarchical regressions) method that allows testing influence of multiple predictor variables in a sequential way [44]. For each of the four mental health outcomes, we, first, fit models that include four family functioning measures as predictors of adolescent mental health; and then add group, time, and group-by-time interaction into the model. Similar to equation (1) above, all models are fit as multilevel mixed-effects models with between-school variability and within-individual variability as a random effect

To examine whether family functioning moderates the intervention effect on adolescent mental health, we add three-way (group-by-time-by family functioning measure) interaction into the equation (1) above. Separate moderator analyses were run for each of the four family functioning measures (i.e., family cohesion, frequency of family communication, comfort of family communication, and child-caregiver relationship). To meet the criteria of temporal precedence of the moderator before the treatment and the independence of the moderator from the intervention [45], we use baseline family functioning measures. Baseline data in the study was collected prior to assignment into treatment conditions, and therefore requirement for temporal precedence of the moderator holds. Independence of moderator from the treatment is tested by fitting the model $M=\eta_{0}+\eta_{1}T+\varepsilon$, where M is the baseline moderator, T is the intervention, and $\eta_{1}=0$ signifies moderator’s independence from the intervention. All four measures of family functioning fit this criterion. To indicate moderator effects, we report joint test of three-way interaction showing interaction of time and group on the slopes of family functioning measure. For family functioning measures showing significant moderator effect, we report adjusted (Sidak’s adjustment) simple effect comparisons (time-within-group at slopes of a continuous moderator) obtained through multiple pairwise comparisons.

## Results

At baseline (Table 1), girls reported moderate scores on all measures of family functioning, including family cohesion (19.6 out of 28), frequency of family communication (13.3 out of 40), comfort of family communication (13.2 out of 30), and quality of child-caregiver relationship (39 out of 56). At baseline, the average depression score reported by girls was 18.5 (out of 63). Girls reported average score of 60.8 (out of 80) on Tennessee Self-Concept Scale, 24 (out of 30) on Rosenberg Self-Esteem Scale, and 4.2 (out of 20) on Beck Hopelessness Scale. These scores align with findings from similar studies conducted among children and adolescents affected by HIV/AIDS and poverty in the region [20,25,26]. The original cutoff points for the Child Depression Inventory, Tennessee Self-Concept Scale, and Beck Hopelessness Scale have been documented elsewhere [35,46,47]. However, acknowledging cross-cultural variations and somatization in the expression of mental health issues, studies have highlighted that cutoff points and diagnostic criteria prescribed by the Diagnostic and Statistical Manual of Mental Disorders often inadequately capture the diversity of mental health experiences across cultures [48,49]. Therefore, we refrain from using these cutoff points in our study. The measures of adolescent mental health employed in our research were culturally adapted to suit vulnerable AIDS-orphaned children and adolescents in Southern Uganda. These measures have been validated in previous studies [21,33], demonstrating strong psychometric properties. In this study, we adopt a perspective that views children’s mental health as a continuum, recognizing that even subclinical deficits in mental health can significantly impact quality of life and raise public health concerns.

Results (Table 2) suggest that receiving the combined treatment (YDA + MFG) significantly improved family cohesion and quality of child-caregiver relationship as reported by adolescent girls. In contrast, the results show no statistically significant effect of the YDA-only treatment on either of these two outcomes. More specifically, compared with their control group counterparts, at 24 months, girls in the YDA + MFG treatment arm reported higher level of family cohesion (EMD^1^ = 1.53; 95% confidence interval (CI) from 0.61 to 2.46; *p* < .001) and better quality of child-caregiver relationship (EMD = 2.33; 95% CI from 0.74 to 3.93; *p* < .001). Further pairwise comparisons also show significant difference between the two treatment arms in terms of intervention effect on family cohesion. Specifically, compared to their counterparts in treatment arm 1 (YDA), at 24 months, adolescent girls in the treatment arm 2 (YDA + MFG) reported higher level of family cohesion (B = 0.9; 95% CI from 0.17 to 1.63; *p* < .01). The results suggest no significant intervention effect, in either treatment arm, on the frequency and comfort of family communication.

Results (Table 3) show that family functioning indicators are strong predictors of adolescent mental health. Results for family cohesion and quality of child-caregiver relationship are consistent across all models. Even after adding group, time, and group-by-time interaction (i.e. treatment effect) into the models, higher scores on family cohesion are significantly associated with lower level of depression (B = −0.17; 95% CI from −0.2 to −0.1; *p* < .001) and hopelessness (B = −0.04; 95% CI from −0.1 to −0.02; *p* < .001), and higher level of self-concept (B = 0.19; 95% CI from 0.1 to 0.3; *p* < .001) and self-esteem (B = 0.05; 95% CI from 0.02 to 0.1; *p* < .01). Similarly, better quality of child-caregiver relationship are significantly associated with lower level of depression (B = −0.22; 95% CI from −0.3 to −0.2; *p* < .001) and hopelessness (B = −0.06; 95% CI from −0.1 to −0.04; *p* < .001), and higher level of self-concept (B = 0.39; 95% CI from 0.3 to 0.4; *p* < .001) and self-esteem (B = 0.08; 95% CI from 0.06 to 0.1; *p* < .01).

In models that include assessment of treatment effect, higher frequency of family communication is significantly associated with higher self-esteem (B = 0.02; 95% CI from 0.005 to 0.04; *p* < .05) and lower hopelessness (B = −0.02; 95% CI from −0.03 to −0.003; *p* < .05). Greater comfort of family communication is significantly associated with lower depression (B = −0.12; 95% CI from −0.2 to −0.1; *p* < .001), reduced hopelessness (B = −0.03; 95% CI from −0.04 to −0.02; *p* < .001), and higher self-concept (B = 0.1; 95% CI from 0.04 to 0.2; *p* < .01).

Moderator analyses and joint test of three-way interaction (Table 4) suggests that effect of the intervention on adolescents’ hopelessness was significantly moderated by family cohesion $\left(\chi^{2}\left(4\right)=21.43;p=\;.000\right)$, frequency of family communication $\left(\chi^{2}\left(4\right)=9.65;p=\;.047\right)$, and quality of child-caregiver relationship $\left(\chi^{2}\left(4\right)=11.12;p=\;.025\right)$. Analyses also suggest that baseline comfort of family communication moderated effect of the intervention on adolescents’ depression $\left(\chi^{2}\left(4\right)=10.2;p=\;.037\right)$.

To better understand the results of moderator analyses, we further examine differences in estimated marginal means across the treatment arms on a slope of continuous moderator (Table 5). Results show that estimated differences of marginal means of Beck Hopelessness Scale between treatment arm 2 (YDA + MFG) and control groups are larger for participants whose baseline score of family cohesion, frequency of family communication, and child-caregiver relations was at 25th percentile. Similarly, estimated differences of means of hopelessness score between treatment arm 1 (YDA) and control group are larger for participants at 25th percentile of baseline family cohesion and child-caregiver relationship score. Results also suggest that estimated differences of means in Beck Depression Scale between both treatment arms and the control group are larger for participants whose baseline score for comfort of family communication was at the 25th percentile. In other words, results of moderation analyses suggest that intervention effect on adolescent mental health was larger for participants who had lower baseline levels of family functioning.

## Discussion

Our study seeks to expand the existing body of knowledge on the relationship between poverty, family dynamics, and mental health of adolescent girls in sub-Saharan Africa. This is one of the few robust evaluations in sub-Saharan Africa examining the impact of an integrated poverty reduction intervention that combines asset accumulation with a separate family strengthening component. Furthermore, our study is one of rare cases examining the impact of an integrated poverty reduction intervention on adolescent mental health while differentiating by family functioning. This differentiation is an important layer in understanding the intersection between poverty, family functioning, and mental health of adolescent girls in families affected by poverty and HIV/AIDS in sub-Saharan Africa.

Research investigating the impact of asset accumulation programs on family functioning is limited, and contrary to few other studies reporting significant effect of asset accumulation interventions on reduced parental stress and improved family communication [23–25], we found no effect of YDA on family functioning. The divergence in our findings from prior studies may be attributed to the demographic composition of our sample, comprised solely of adolescent girls. Existing research points to gender differences in how adolescents engage with their caregivers and report family cohesion, support and communication [50,51]. Therefore, our findings suggest the need for research that examines gender-specific differences in the effect of YDA on family functioning reported by adolescents.

This being said, we found significant improvement in family cohesion and quality of child-caregiver relationship among participants who received asset accumulation intervention combined with the MFG component. These findings are consistent with the scarce evidence from other integrated interventions suggesting that poverty reduction module combined with a separate family strengthening component may significantly improve parenting practices in poor households [27]. Our findings also align with previous studies showing significant positive effect of MFG interventions on parent-adolescent relationship, family cohesion, improved family dynamics, and family functioning [18,52].

Our findings demonstrate a significant positive association between family functioning—such as family cohesion, frequency and comfort of family communication and child-caregiver relationship—and mental health of adolescent girls in our sample. Moreover, we found that family functioning moderated the effect of the intervention on the Beck Hopelessness Scale and Depression. Specifically, results of our moderation analyses strongly suggest that the intervention was most effective in reducing score on Beck Hopelessness Scale for girls who came from families with lower levels of family functioning at the start of the intervention. Additionally, our findings suggest that combining the asset accumulation opportunity with the MFG component had a stronger effect on reducing adolescent depression than providing the asset accumulation opportunity alone. These findings are in line with the Family Stress Model [8,10] and consistent with a number of previous studies that indicate the crucial role of family functioning as a protective factor mitigating effects of poverty on mental health of adolescents [11,13,14].

Our findings add to the scarce body of knowledge emphasizing the importance of interventions that combine asset accumulation and poverty reduction opportunities with a separate family strengthening component to help families build resilience and address the emotional and psychological stressors caused by poverty. These interventions provide families with economic opportunities—thus potentially reducing the effect of economic stressors on family functioning and adolescent mental health—as well as supportive space and skills to strengthen family relations and enhance family resilience.

### Limitations

The study has several limitations to be acknowledged. First, the generalizability of the findings may be limited as the sample consisted exclusively of girls enrolled in secondary schools, potentially biasing the results towards those with higher socioeconomic status and better mental health. However, it is important to emphasize that the primary focus of this study was prevention, targeting girls in schools. While recognizing the significance of providing support beyond school settings, including street children and those who have dropped out, this study was designed within a prevention framework to assist vulnerable adolescent girls before they drop out of school when their exposure to HIV-risk taking behaviors increases [31]. For future research, it is crucial to explore comprehensive interventions that consider the intersection of poverty and mental health while addressing the needs of diverse subgroups within the population of adolescent girls.

Another limitation is the reliance on self-report measures. The absence of biomarkers or objective measures of mental health and family functioning may introduce limitations, as self-report data are susceptible to biases such as social desirability. Acknowledging these limitations, it is important to note that implementing objective evaluations of adolescent mental health and family functioning through assessments conducted by trained clinicians presents a significant challenge in low-income settings, such as Uganda. This challenge stems from the well-documented scarcity of health and social services, including a low concentration of trained mental health professionals and family clinicians, which is reflective of the broader systemic gaps in health infrastructure [53,54]. Future research would benefit from incorporating additional objective measures as well as the social desirability scales to detect, control for, and mitigate potential biases and enhance the validity of the results.

Furthermore, it is important to acknowledge that the study focused on adolescent girls, and no data were collected from parents or caregivers. Incorporating parental perspectives in the assessment of family dynamics could offer valuable insights into the broader understanding of adolescent mental health and family functioning.

## Supplementary Material

Consort Flow Diagram

## Figures and Tables

**Table 1:** Baseline characteristics of the study sample

Variables	Control group (n = 408)		Treatment arm 1 (n = 471)		Treatment arm 2 (n = 381)		Total (n = 1,260)		Adjusted wald test
					
	Percentage or mean [95% confidence intervals]								

Family functioning									
Family cohesion (range: 0–28)	19.8	[18.8; 20.7]	19.3	[18.5; 20.1]	19.7	[19.2; 20.2]	19.6	[19.1; 20]	F (2, 45) = 0.44; *p* = .6
Frequency of family communication (0–40)	12.7	[12.02; 13.4]	13.9	[13.1; 14.7]	13.2	[12.3; 14.2]	13.3	[12.8; 13.8]	F (2, 45) = 2.4; *p* = .1
Comfort of family communication (0–30)	13.1	[12.2; 13.9]	13.4	[12.7; 14.2]	13	[12.4; 13.6]	13.2	[12.8; 13.6]	F (2, 45) = 0.42; *p* = .6
Child-caregiver relationship (range: 0–56)	39	[37.8; 40.2]	29	[37.8; 40.2]	39.4	[38.2; 50.6]	39.1	[38.4; 39.8]	F (2, 45) = 0.13; *p* = .9
Child mental health									
Beck depression inventory (range: 0–63)	19.2	[18.1; 20.3]	17.9	[16.8; 18.9]	18.5	[17.3; 19.6]	18.5	[17.8; 19.1]	F (2, 45) = 1.5; *p* = .2
Tennessee self-concept scale (range: 0–80)	60.6	[59.1; 62.1]	61.1	[59.8; 62.5]	60.7	[59.7; 61.7]	60.8	[60.1; 61.6]	F (2, 45) = 0.16; *p* = .8
Rosenberg self-esteem scale (Range: 0–30)	23.9	[23.5; 24.4]	24.5	[24; 24.9]	23.6	[23.1; 23.99]	24	[23.7; 24.3]	F (2, 45) = 4.47*; *p* = .02
Beck hopelessness Scale (Range: 0–20)	4.1	[3.8; 4.4]	4.2	[3.8; 4.5]	4.3	[4; 4.7]	4.2	[4; 4.4]	F (2, 45) = 0.59; *p* = .6

**Boldface** type indicates statistically significant results.

*** *p* < .001

** *p* < .01

* *p* < .05.

We report adjusted Wald F-statistics (**Design-based F**) to examine individual-level variations while accounting for potential correlation between same-school observations.

**Table 2:** Effect of the intervention on family functioning

Outcomes	Family cohesion		Frequency of family communication		Comfort of family communication		Child-caregiver relationship	
				
	EMD^a^	95% CI	EMD^a^	95% CI	EMD^a^	95% CI	EMD^a^	95% CI

Treatment arm 1^b^ versus control arm								
At baseline	−0.50	[−1.84; 0.84]	1.13	[−0.13; 2.39]	0.42	[−0.89; 1.74]	−0.18	[−2.05; 1.68]
At 12 months	0.35	[−0.98; 1.69]	−1.14	[−2.91; 0.64]	−0.66	[−2.10; 0.78]	0.73	[−1.27; 2.72]
At 24 months	0.63	[−0.29; 1.55]	0.37	[−1.29; 2.02]	0.11	[−1.35; 1.58]	0.61	[−0.97; 2.19]
Treatment arm 2^c^ versus control arm								
At baseline	−0.06	[−1.29; 1.17]	0.50	[−0.91; 1.92]	0.09	[−0.11; 1.34]	0.43	[−1.61; 2.47]
At 12 months	0.79	[−0.49; 2.07]	−0.80	[−2.59; 0.99]	−0.35	[−1.92; 1.21]	1.43	[−0.40; 3.26]
At 24 months	**1.53** ***	[0.61; 2.46]	−0.21	[−1.90; 1.49]	0.12	[−1.43; 1.68]	**2.33** ***	[0.74; 3.93]
Treatment arm 2 versus treatment arm 1								
At baseline	0.44	[−0.59; 1.47]	−0.63	[−2.11; 0.85]	−0.34	[1.02; 0.71]	0.62	[−1.31; 2.55]
At 12 months	0.44	[−0.89; 1.77]	0.33	[−1.48; 2.15]	0.30	[−1.23; 1.84]	0.70	[−1.29; 2.70]
At 24 months	**0.90** **	[0.17; 1.63]	−0.57	[−2.22; 1.08]	0.01	[−1.43; 1.45]	1.72	[−0.12; 3.57]
Joint test of interaction effect	χ^2^ (4) = 13.06*; *p* = .011		χ^2^ (4) = 9.38; *p* = .052		χ^2^ (4) = 4.36; *p* = .359		χ^2^ (4) = 11.07*; *p* = .026	
Observations	3,644		3,644		3,644		3,644	
Number of groups	47		47		47		47	

Boldface type indicates statistically significant results.

*** *p* < .001

** *p* < .01

* *p* < .05.

a EMD is differences of estimated marginal means. These coefficients represent group-within-time simple effects.

b Treatment arm 1 (YDA) offered enrollment in a savings program (Youth Development Accounts) with a 1:1 savings match. Access to the matching funds required completion of 12 workshops covering basic principles of financial management, held over 12 months by the community agency in partnership with YDA holding banks. The matched funds aimed to support costs of adolescent’s education and skill training.

c Treatment arm 2 (YDA + MFG) provided a 16-week multifamily group (MFG) family-strengthening intervention alongside YDA. Trained community health workers and peer parents delivered a weekly curriculum, focusing on the 4 Rs (Rules, Responsibility, Relationships, and Respectful Communication) and 2Ss (Stress and Social Support). Topics included behavioral health, support system development, enhancing family communication, and resource mobilization for environmental stressors. The intervention was tailored to the context of Uganda.

**Table 3:** Family functioning as predictor of adolescent mental health

Variables	Beck’s depression inventory		Tennessee self-concept scale		Rosenberg self-esteem scale		Beck hopelessness scale	
				
	Beta-coefficient	95% CI	Beta-coefficient	95% CI	Beta-coefficient	95% CI	Beta-coefficient	95% CI

Model without intervention								
Family functioning								
Family cohesion	**−0.20** ***	[−0.3; −0.1]	**0.21** ***	[0.1; 0.3]	**0.06** ***	[0.03; 0.1]	**−0.05** ***	[−0.1; −0.02]
Frequency of family communication	**−0.04** *	[−0.1; −0.01]	0.00	[−0.1; 0.1]	**0.04** ***	[0.02; 0.1]	**−0.02** **	[−0.03; −0.005]
Comfort of family communication	**−0.13** ***	[−0.2; −0.1]	**0.10** **	[0.04; 0.2]	0.02	[−0.01; 0.04]	**−0.03** ***	[−0.04; −0.02]
Child-caregiver relationship	**−0.19** ***	[−0.2; −0.1]	**0.38** ***	[0.3; 0.4]	**0.06** ***	[0.04; 0.1]	**−0.05** ***	[−0.1; −0.04]
Constant	28.69***	[27.0; 30.4]	42.33***	[40.5; 44.1]	20.89***	[20.1; 21.7]	7.28***	[6.8; 7.7]
Observations	3,644		3,409		3,359		3,644	
Number of groups	47		47		47		47	
Model with intervention								
Family functioning								
Family cohesion	**−0.17** ***	[−0.2; −0.1]	**0.19** ***	[0.1; 0.3]	**0.05** **	[0.02; 0.1]	**−0.04** ***	[−0.1; −0.02]
Frequency of family communication	−0.02	[−0.0; 0.0]	−0.01	[−0.1; 0.1]	**0.02** *	[0.005; 0.04]	**−0.02** *	[−0.03; −0.003]
Comfort of family communication	**−0.12** ***	[−0.2; −0.1]	**0.10** **	[0.04; 0.2]	0.01	[−0.0; 0.0]	**−0.03** ***	[−0.04; −0.02]
Child-caregiver relationship	**−0.22** ***	[−0.3; −0.2]	**0.39** ***	[0.3; 0.4]	**0.08** ***	[0.06; 0.1]	**−0.06** ***	[−0.1; −0.04]
Treatment arm 1^b^ versus control arm^a^								
At 12 months	**−2.08**	[−3.35; −0.81]	**1.96**	[0.34; 3.58]	0.53	[−0.17; 1.23]	**−0.37**	[−0.74; −0.003]
At 24 months	−1.04	[−2.29; 0.20]	1.59	[0.07; 3.25]	0.14	[−0.43; 0.72]	−0.29	[−0.71; 0.12]
Treatment arm 2^c^ versus control arm^a^								
At 12 months	**−2.47**	[−3.93; −1.02]	1.25	[1.04; 3.53]	0.56	[−0.10; 1.22]	**−0.30**	[−0.57; −0.02]
At 24 months	**−2.00**	[−3.20; −0.81]	1.65	[0.29; 3.59]	0.17	[−0.50; 0.84]	−0.36	[−0.80; −0.09]
Treatment arm 2 versus treatment arm 1^a^								
At 12 months	−0.39	[−1.60; 0.83]	−0.72	[2.74; 1.31]	0.03	[−0.60; 0.67]	0.07	[−0.25; 0.39]
At 24 months	−0.96	[−2.28; 0.37]	0.06	[1.65; 1.77]	0.03	[−0.49; 0.54]	−0.07	[−0.41; 0.28]
Constant	32.74***	[30.8; 34.7]	40.42***	[38.1; 42.8]	19.32***	[18.5; 20.2]	7.69***	[7.1; 8.2]
Observations	3,644		3,409		3,359		3,644	
Number of groups	47		47		47		47	

Boldface type indicates statistically significant results.

*** *p* < .001

** *p* < .01

* *p* < .05.

a EMD is differences of estimated marginal means. These coefficients represent group-within-time simple effects.

b Treatment Arm 1 (YDA) offered enrollment in a savings program (Youth Development Accounts) with a 1:1 savings match. Access to the matching funds required completion of 12 workshops covering basic principles of financial management, held over 12 months by the community agency in partnership with YDA holding banks. The matched funds aimed to support costs of adolescent’s education and skill training.

c Treatment Arm 2 (YDA + MFG) provided a 16-week multifamily group (MFG) family-strengthening intervention alongside YDA. Trained community health workers and peer parents delivered a weekly curriculum, focusing on the 4 Rs (Rules, Responsibility, Relationships, and Respectful Communication) and 2Ss (Stress and Social Support). Topics included behavioral health, support system development, enhancing family communication, and resource mobilization for environmental stressors. The intervention was tailored to the context of Uganda.

**Table 4:** Family functioning as moderator: Joint test of three-way interaction

Moderator	Beck’s depression inventory		Tennessee self-concept scale		Rosenberg self-esteem scale		Beck hopelessness scale	
				
	χ^2^ (4)	*p* > χ^2^ (4)	χ^2^ (4)	*p* > χ^2^ (4)	χ^2^ (4)	*p* > χ^2^ (4)	χ^2^ (4)	*p* > χ^2^ (4)

Family functioning								
Family cohesion	0.87	.929	0.32	.989	1.33	.856	**21.43** ***	.000
Frequency of family Communication	9.35	.053	8.31	.081	3.25	.516	**9.65** *	.047
Comfort of family communication	**10.2** *	.037	8.76	.067	1.91	.752	8.20	.085
Child-caregiver relationship	1.42	.842	1.63	.804	4.40	.354	**11.12** *	.025
Observations	3,644		3,409		3,359		3,644	
Number of groups	47		47		47		47	

Boldface type indicates statistically significant results.

*** *p* < .001

** *p* < .01

* *p* < .05.

**Table 5:** Effect of the intervention on adolescent mental health moderated by family functioning

Moderator variables	Depression moderated by comfort of family communication		Hopelessness moderated by family cohesion		Hopelessness moderated by frequency of family communication		Hopelessness moderated by child-caregiver relationship	
				
	EMD^a^	95% CI	EMD^a^	95% CI	EMD^a^	95% CI	EMD^a^	95% CI

Moderator at 25th percentile								
Treatment arm 1^b^ versus control arm								
At baseline	−0.88	[−3.12; 1.35]	0.17	[−0.42; 0.76]	0.13	[−0.45; 0.71]	0.17	[−0.51; 0.84]
At 12 months	−1.21	[−3.09; 0.67]	**−0.57**	[−1.03; −0.11]	−0.44	[−0.92; 0.04]	**−0.56**	[−1.07; −0.06]
At 24 months	−1.01	[−2.78; 0.77]	−0.47	[−1.03; 0.09]	−0.31	[−0.82; 0.20]	−0.39	[−0.87; 0.09]
Treatment arm 2^c^ versus control arm								
At baseline	−0.64	[−2.68; 1.40]	0.57	[−0.06; 1.20]	0.29	[−0.23; 0.81]	0.62	[−0.02; 1.26]
At 12 months	**−3.34**	[−4.97; −1.72]	**−0.53**	[−0.94; −0.12]	**−0.47**	[−0.85; −0.09]	**−0.53**	[−0.99; −0.07]
At 24 months	**−3.67**	[−5.73; −1.61]	**−0.73**	[−1.34; −0.11]	**−0.71**	[−1.22; −0.20]	**−0.64**	[−1.20; −0.09]
Treatment arm 2 versus treatment arm 1								
At baseline	0.24	[−1.81; 2.30]	0.40	[−0.25; 1.06]	0.16	[−0.46; 0.78]	0.45	[−0.19; 1.09]
At 12 months	**−2.14**	[−4.06; −0.21]	0.04	[−0.44; 0.52]	−0.03	[−0.45; 0.40]	0.03	[−0.46; 0.52]
At 24 months	**−2.66**	[−4.70; −0.62]	−0.25	[−0.72; 0.21]	−0.40	[−0.87; 0.07]	−0.25	[−0.72; 0.22]
Moderator at 50th percentile								
Treatment arm 1 versus control arm								
At baseline	−1.16	[−2.95; 0.62]	0.01	[−0.52; 0.54]	0.14	[−0.40; 0.67]	0.05	[−0.45; 0.55]
At 12 months	**−1.96**	[−3.57; −0.36]	**−0.42**	[−0.80; −0.03]	−0.36	[−0.82; 0.09]	−0.37	[−0.79; 0.04]
At 24 months	−1.23	[−2.50; 0.04]	−0.39	[−0.83; 0.04]	−0.34	[−0.79; 0.12]	−0.37	[−0.81; 0.07]
Treatment arm 2 versus control arm								
At baseline	−0.73	[−2.49; 1.02]	0.22	[−0.28; 0.71]	0.27	[−0.25; 0.79]	0.23	[−0.25; 0.71]
At 12 months	**−2.98**	[−4.60; −1.35]	**−0.37**	[−0.69; −0.04]	−0.34	[−0.73; 0.04]	**−0.34**	[−0.68; −0.005]
At 24 months	**−2.97**	[−4.52; −1.41]	**−0.53**	[−1.00; −0.06]	**−0.48**	[−0.96; −0.004]	**−0.52**	[−0.98; −0.06]
Treatment arm 2 versus treatment arm 1								
At baseline	0.43	[−1.41; 2.27]	0.21	[−0.33; 0.75]	0.13	[−0.41; 0.68]	0.19	[−0.32; 0.69]
At 12 months	−1.01	[−2.57; 0.54]	0.05	[−0.31; 0.41]	0.02	[−0.37; 0.42]	0.03	[−0.35; 0.41]
At 24 months	**−1.73**	[−3.32; −0.14]	−0.14	[−0.51; 0.24]	−0.15	[−0.54; 0.25]	−0.15	[−0.53; 0.24]
Moderator at 75th percentile								
Treatment arm 1 versus control arm								
At baseline	−1.45	[−3.10; 0.21]	−0.15	[−0.76; 0.46]	0.16	[−0.42; 0.73]	−0.06	[−0.60; 0.48]
At 12 months	**−2.72**	[−4.39; −1.04]	−0.27	[−0.80; 0.27]	−0.22	[−0.84; 0.39]	−0.21	[−0.70; 0.29]
At 24 months	**−1.46**	[−2.72; −0.20]	−0.32	[−0.81; 0.18]	−0.38	[−1.00; 0.24]	−0.35	[−0.97; 0.26]
Treatment arm 2 versus control arm								
At baseline	−0.83	[−2.58; 0.93]	−0.14	[−0.69; 0.41]	0.24	[−0.41; 0.89]	−0.11	[−0.64; 0.42]
At 12 months	**−2.61**	[−4.63; −0.58]	−0.20	[−0.73; 0.32]	−0.12	[−0.69; 0.45]	−0.18	[−0.59; 0.23]
At 24 months	**−2.27**	[−3.67; −0.86]	−0.33	[−0.91; 0.24]	−0.09	[−0.72; 0.54]	−0.41	[−0.99; 0.18]
Treatment arm 2 versus treatment arm 1								
At baseline	0.62	[−1.21; 2.45]	0.01	[−0.57; 0.59]	0.09	[−0.48; 0.66]	−0.05	[−0.60; 0.50]
At 12 months	0.11	[−1.51; 1.73]	0.06	[−0.39; 0.51]	0.10	[−0.43; 0.64]	0.03	[−0.51; 0.56]
At 24 months	−0.80	[−2.20; 0.59]	−0.02	[−0.49; 0.46]	0.29	[−0.15; 0.74]	−0.05	[−0.60; 0.49]
Observations	3,644		3,644		3,644		3,644	
Number of groups	47		47		47		47	

Boldface type indicates statistically significant results.

*** *p* < .001

** *p* < .01

* *p* < .05.

a EMD is differences of estimated marginal means. These coefficients represent group-within-time simple effects.

b Treatment arm 1 (YDA) offered enrollment in a savings program (Youth Development Accounts) with a 1:1 savings match. Access to the matching funds required completion of 12 workshops covering basic principles of financial management, held over 12 months by the community agency in partnership with YDA holding banks. The matched funds aimed to support costs of adolescent’s education and skill training.

c Treatment arm 2 (YDA + MFG) provided a 16-week multifamily group (MFG) family-strengthening intervention alongside YDA. Trained community health workers and peer parents delivered a weekly curriculum, focusing on the 4 Rs (Rules, Responsibility, Relationships, and Respectful Communication) and 2 Ss (Stress and Social Support). Topics included behavioral health, support system development, enhancing family communication, and resource mobilization for environmental stressors. The intervention was tailored to the context of Uganda.

## Footnotes

1 To report the treatment effect, we report time-within-group simple effect comparisons obtained through multiple pairwise comparisons. EMD is differences of estimated marginal means.
