# Supplementary material for: Combining Asset Accumulation and Multifamily Group Intervention to Improve Mental Health for Adolescent Girls: A Cluster-Randomized Trial in Uganda
Source: J Adolesc Health. Author manuscript; Available in PMC 2024 Feb 5. (PMC10840800; doi:10.1016/j.jadohealth.2023.08.012)
Supplement: Consort Flow Diagram [file NIHMS1932006-supplement-Consort_Flow_Diagram.pdf]

Consort flow chart

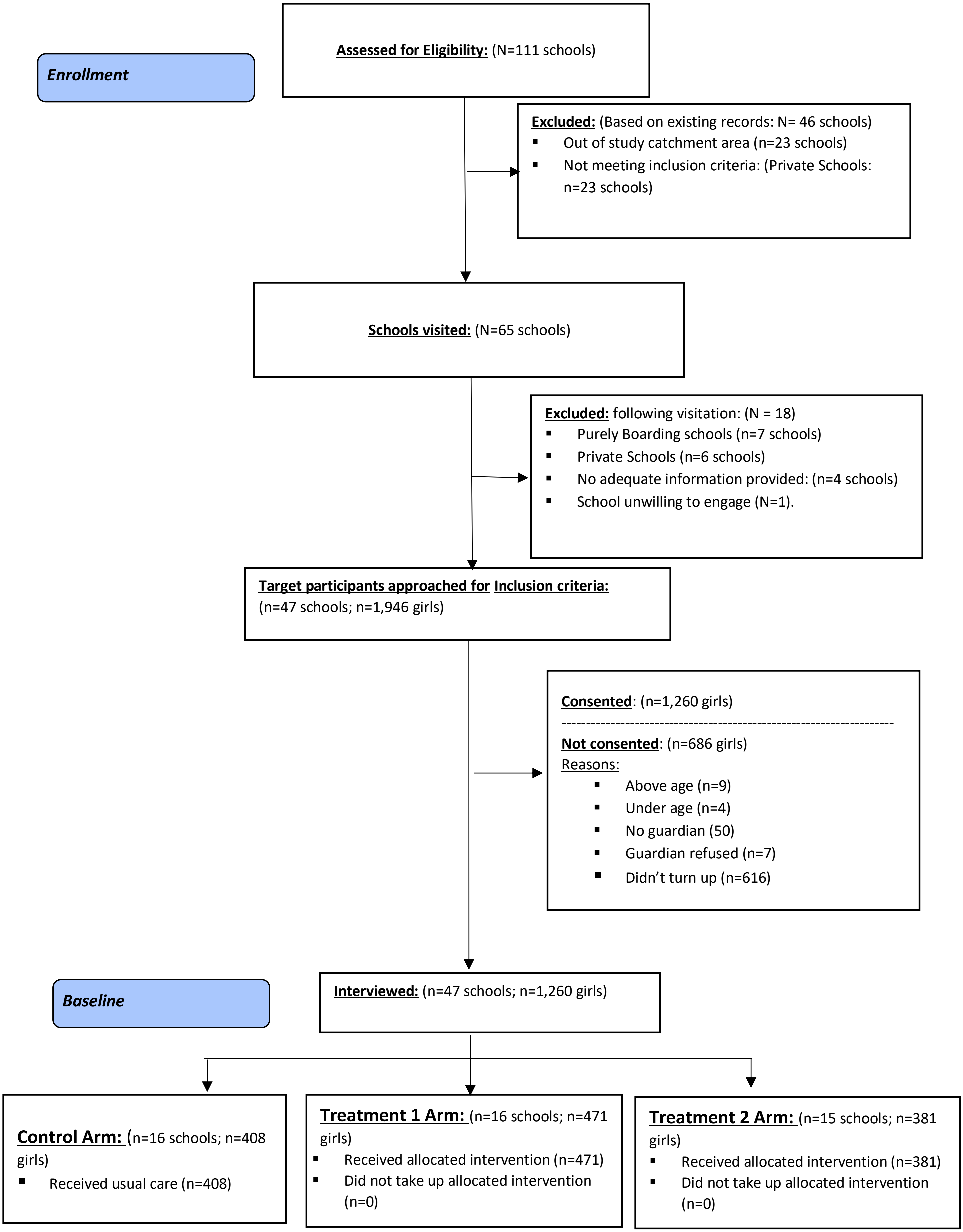

Follow-up: 12 - Months

12-month (Wave 2): (n=16 schools; n=396 girls)

- Lost to follow-up (n=12); Moved out of study area, or could not be traced.

12-month (Wave 2): (n=16 schools; n=457 girls)

- Lost to follow-up (n=13); Moved out of study area, or could not be traced.
- Unwillingness to continue participating in the study (n=1)

12-month (Wave 2): (n=15 schools; n=366 girls)

- Lost to follow-up (n=15); Moved out of study area, or could not be traced.

Follow-up: 24 - Months

24-month (Wave 3): (n=16 schools; n=380 girls)

- Lost to follow-up (n=28); Moved out of study area, or could not be traced.

24-month (Wave 3): (n=16 schools; n=441 girls)

- Lost to follow-up (n=29); Moved out of study area, or could not be traced.
- Unwillingness to continue participating in the study (n=1)

24-month (Wave 3): (n=15 schools; n=344 girls)

- Lost to follow-up (n=36); Moved out of study area, or could not be traced.
- Died (n=1)
